# P-2008. Knowledge, Attitudes, and Practices Regarding a Multiplex PCR System for Rapid Identification of Pathogens in Bloodstream Infections: A Cross-sectional Study Among Infectious Diseases Trainees in a Tertiary Care Centre in North India

**DOI:** 10.1093/ofid/ofaf695.2172

**Published:** 2026-01-11

**Authors:** Md Tariq Maula, Sandeep Rao Kordcal, Piyush Ranjan

**Affiliations:** All India Institute of Medical Sciences, New Delhi, Delhi, India; All India Institute of Medical Sciences, New Delhi, Delhi, India; All India Institute of Medical Sciences, New Delhi, New Delhi, Delhi, India

## Abstract

**Background:**

The BioFire FilmArray Blood Culture Identification 2 (BCID2) panel allows for rapid detection of bloodstream pathogens and key resistance genes directly from flagged blood culture bottles in suspected bloodstream infections (BSIs). While its diagnostic accuracy has been well established, meaningful clinical impact depends on how confidently and consistently it is applied at the bedside. This study explored the knowledge, attitudes, and practices (KAP) of infectious diseases (ID) trainees toward BCID2 use in a high-burden tertiary care centre.

**Table 1:**
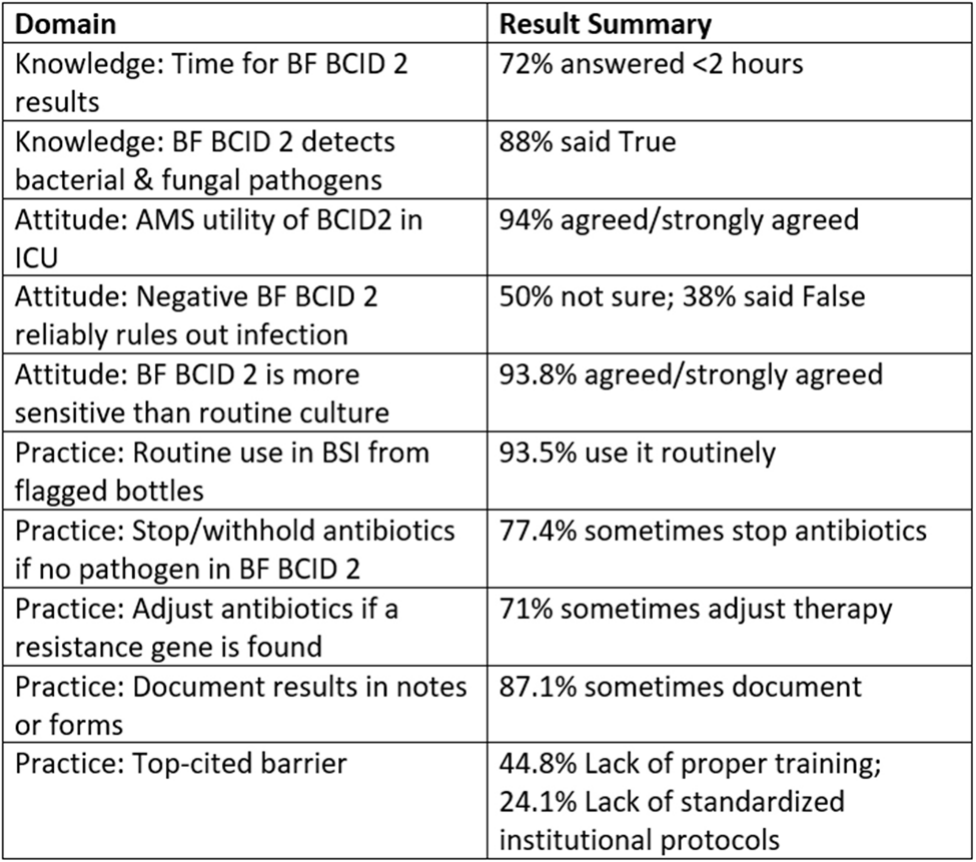
Summary of the important KAP questions and responses for the use of BCID2 in the diagnosis of BSI

**Figure 1:**
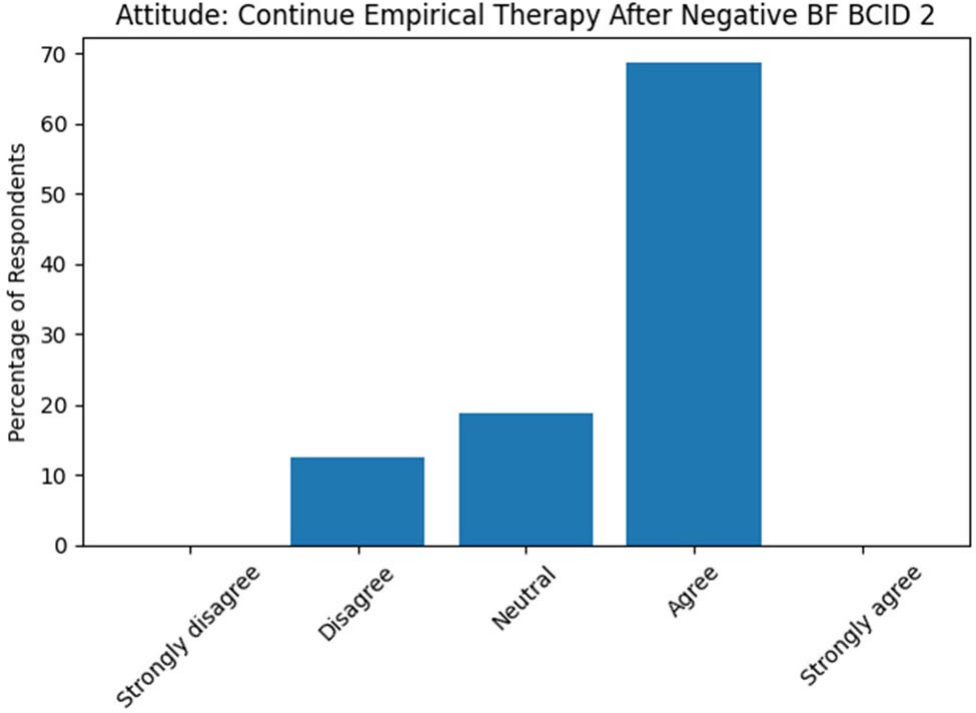
68.8% of respondents agreed that they preferred to continue empirical antimicrobial therapy when BCID 2 results were negative

**Methods:**

A structured 25-item anonymized KAP questionnaire was administered to ID resident doctors at a tertiary care centre in North India. The survey assessed BCID2 test awareness and interpretation of results, confidence in antimicrobial decisions based on test results, frequency of routine use, and potential barriers to full utilization. Descriptive analyses were performed on 32 completed responses.

**Results:**

87.5% of respondents correctly identified that BCID2 detects both bacterial and fungal pathogens, and 71.9% were aware of its rapid turnaround time (< 2 hours). However, a substantial number of respondents incorrectly identified *Burkholderia cepacia* (45.2%) and *Aspergillus fumigatus* (41.9%) to be part of the panel, signifying gaps in knowledge. Most participants (93.5%) reported using BCID2 routinely for BSIs, and 90.3% adjusted therapy based on resistance markers in the appropriate clinical context. 62.5% agreed and 28.1% strongly agreed that conventional culture remains essential even after negative BCID 2. Clinical severity and BCID2 results were the top influences on antibiotic choice (45.2% and 41.9%, respectively) in BSI. The majority of respondents agreed (34.4%) or strongly agreed (59.4%) that BCID2 improves antimicrobial stewardship (AMS) in ICU settings. Lack of proper training (44.8%) and lack of standardised institutional protocols (24.1%) were cited as the biggest barriers to its use.

**Conclusion:**

BCID2 is widely adopted by ID trainees in clinical practice, however, gaps persist in interpretation and integration into standardised protocols. Targeted training programs and incorporation into standard practice protocols are needed to enhance the impact of rapid diagnostics on antimicrobial stewardship.

**Disclosures:**

All Authors: No reported disclosures

